# Outcomes of COVID-19 in Patients with Mental Disorders

**DOI:** 10.1192/j.eurpsy.2022.1273

**Published:** 2022-09-01

**Authors:** N. Petrova, M. Sivashova, V. Pashkovsky, G. Prokopovich, A. Gvozdetckii

**Affiliations:** 1 Saint-Petersburg University, Department Of Psychiatry And Addiction, Saint-Petersburg, Russian Federation; 2 St. Petersburg State University, Department Of Psychiatry And Narcology, St. Petersburg, Russian Federation; 3 North-Western State Medical University named after I.I. Mechnikov, Department Of Psychiatry And Addiction, Saint-Petersburg, Russian Federation

## Abstract

**Introduction:**

Clinical practice has shown that SARS-CoV-2 viral infection increases the likelihood of developing mental disorders.Clinical practice has shown that SARS-CoV-2 viral infection increases the likelihood of developing mental disorders.

**Objectives:**

To analyze clinical indicators of patients with COVID-19 with mental disorders and to identify predictors of adverse outcomes associated with mental state on its basis.

**Methods:**

The study included 97 patients, 41 men and 56 women (62.3±15.3 years of age). During the observation period, 26 people died and 71 people recovered. Data collection was carried out using a questionnaire (109 variables). Binary logistic regression and Cox proportional hazards regression were used.

**Results:**

In the study group, death occurred on average after 11.5 days. In this group, the mental state of patients was more severe with a predominance of cases of delirium. With age, the probability of a fatal outcome increased by 1.03 with each year of life. The severity of mental disorder had a greater impact on the risk of death compared to age (p=0.003). Improvement of the mental state of patients during psychotropic therapy was associated with a reduction in the risk of an unfavorable outcome of coronavirus infection by 11.11 times. The greatest contribution to the unfavorable outcome was made by the severity of infection: the risk of death increased by 33.17 times.

**Conclusions:**

A severe or extremely severe mental state increased the risk of death by 4.55 times. The most significant factor in predicting mortality was associated with the severity of the underlying disease.

**Disclosure:**

No significant relationships.

